# Multidrug-Resistant *Mycobacterium tuberculosis* in a Community Hospital, Luanda, Angola

**DOI:** 10.3201/eid3103.241831

**Published:** 2025-03

**Authors:** Ngiambudulu M. Francisco, Alberto Gaviraghi, Francesca Alladio, Ralph Huits, Bruna Carnielli, Elena Salvador, Giacomo Stroffolini, Niccolò Ronzoni, Paolo Cattaneo, Concetta Castilletti, Alberto Kalume, Cristina Mazzi, Alberto Matteelli, Dora Buonfrate, Federico G. Gobbi

**Affiliations:** Instituto Nacional de Investigação em Sáude, Luanda, Angola (N.M. Francisco); IRCCS Ospedale Sacro Cuore Don Calabria, Negrar, Italy (A. Gaviraghi, F. Alladio, E. Salvador, G. Stroffolini, N. Ronzoni, P. Cattaneo, C. Castilletti, C. Mazzi, D. Buonfrate, F.G. Gobbi); IRCCS Sacro Cuore Don Calabria Hospital, Verona, Italy (R. Huits); Presidio Ospedale Santa Maria del Prato, Feltre, Italy (B. Carnielli); Hospital Divina Providencia, Luanda (A. Kalume); University of Brescia and Brescia Spedali Civili General Hospital WHO Collaborating Centre for TB/HIV and TB Elimination, Brescia, Italy (A. Matteelli); University of Brescia, Brescia (F.G. Gobbi)

**Keywords:** tuberculosis and other mycobacteria, bacteria, Mycobacterium tuberculosis, antimicrobial resistance, multidrug resistance, MDR, pre-XDR, Angola

## Abstract

In a longitudinal study in a first-level hospital in Luanda, Angola, we found rifampin-resistant and multidrug-resistant tuberculosis (TB) in 38 (8%, 95% CI 5.7–10.8) of 474 patients with no previous history of TB. Of note, 2 patients (0.4%, 95% CI 0.1–1.5) demonstrated pre–extensively drug-resistant TB.

Tuberculosis (TB) is one of the leading causes of death worldwide, causing an estimated 1.25 million deaths in 2023 (95% uncertainty interval [UI] 1.13–1.37 million) (*1*,*2*). Drug resistance is a major threat to the effective treatment of TB; rifampin-resistant (RR) and multidrug-resistant (MDR; resistance to both rifampin and isoniazid) TB caused an estimated 150,000 (95% UI 94,000–210,000) deaths in 2023 (*2*). Even more worrisome is the emergence of pre–extensively drug-resistant (pre-XDR; resistance to rifampin and any fluoroquinolone) and extensively drug-resistant (XDR TB; resistance to rifampin, any fluoroquinolone, and >1 of bedaquiline or linezolid) strains, which entail the deployment of more expensive and less tolerated drugs (*2*).

Angola is one of 30 countries with high TB burden; ≈22,000 (95% UI 14,000–32,000) deaths were caused by TB in 2023 (*2*,*3*). The incidence rate in the country is 339 cases/100,000 population (95% UI 217–511 cases/100,000 population), which is still far from the 50% incidence rate reduction target set for 2015 (when the rate was 366 cases/100,000 population [95% UI 232–531 cases/100,000 population]) to 2025 by the End TB strategy (*2*,*4*). However, the official data on TB in Angola are based on notifications only; thus, the World Health Organization (WHO) encourages nationwide surveys to collect more reliable data (*2*,*5*).

During November 21, 2023–June 14, 2024, we conducted a study to determine the prevalence of drug-resistant TB at the Hospital Divina Providência, a first-level hospital in the district of Kilamba-Kiaxi, Luanda, Angola. TB cases were diagnosed with GeneXpert Ultra assay (Cepheid, https://www.cepheid.com). Patients who tested positive for RR or MDR TB were referred to the Centro Especializado de Tratamento de Endemias e Pandemias (CETEP) for further management, in accordance with national guidelines. The Ministry of Health Research Ethics Committee, Angola, approved the study (ref. 32/C.E.M.S/2023). 

We calculated sample size using a conservative percentage of MDR TB and RR-TB of 20%. We calculated a 2-sided 95% CI with a width of 10% for >264 TB-infected participants. We measured potential associations between resistance and demographic and clinical variables. We summarized continuous variables by median and interquartile range and categorical variables by number and percentage. We used exact CIs for CI proportions. We calculated odds ratios (ORs) with 95% CIs to identify possible risk factors (age, sex, body mass index, smoking status, alcohol consumption, and HIV infection) associated with *Mycobacterium tuberculosis* infection, RR-MTB, and MDR TB in multivariable logistic regression models. We used R version 4.4.1 (The R Project for Statistical Computing, https://www.r-project.org) for all analyses.

During the study period, 474 cases of TB were confirmed in patients with no history of TB. We detected RR-TB in 38 (8%, 95% CI 5.7%–10.8%) and MDR TB in 19 (4.0%, 95% CI 2.4%–6.2%) of the 474 cases. Of note, 2 cases (0.4%, 95% CI 0.1%–1.5%) showed resistance also to quinolones (pre-XDR TB). No cases of XDR TB were detected (Table 1). None of the considered variables was associated with an increased risk for RR or MDR TB (Table 2). 

**Table 1:** Resistance profiles of cases in study of multidrug-resistant tuberculosis, Luanda, Angola

Drug	No. (%) cases showing resistance, N = 474
Rifampin	38 (8.0)
Isoniazid	19 (4.0)
Fluoroquinolones	2 (0.4)
Amikacin	0
Ethionamide	4 (0.8)

**Table 2:** Multivariable logistic regression models evaluating risk factors for RR and MDR TB, Luanda, Angola*

Characteristic	RR TB			MDR TB	
	OR (95% CI)	p value		OR (95% CI)	p value
Age	1 (0.97–1.03)	0.8		1.02 (0.98–1.06)	0.4
Sex					
M	Referent			Referent	
F	0.59 (0.27–1.25)	0.2		0.39 (0.13–1.07)	0.075
Body mass index	0.96 (0.84–1.08)	0.5		0.91 (0.75–1.07)	0.3
Smoking					
N	Referent			Referent	
Y	0.84 (0.22–2.63)	0.8		0.44 (0.02–2.85)	0.5
Alcohol					
N	Referent			Referent	
Y	1.01 (0.38–2.46)	>0.9		0.85 (0.18–3.01)	0.8
HIV					
Negative	Referent			Referent	
Positive	1.16 (0.26–3.76)	0.8		0.57 (0.03–3.23)	0.6

*MDR, multidrug-resistant; OR, odds ratio; RR, rifampin-resistant; TB, tuberculosis.

In our study, the prevalence of RR or MDR TB in patients with no previous history of TB was at least double the estimates reported by WHO for 2023 in Angola (3.29%–3.65%) (*3*). Similarly, a previous study in a rural area (Cubal, in Benguela Province) in 2014 reported prevalence of MDR TB as twice as high as estimates: 8% (95% CI 5.1%–12.3%) (*6*). In 2014, a study carried out at Hospital Divina Providência found 5.6% of RR/MDR TB in 89 patients who were tested for TB (no distinction between new and previously treated cases) (*7*). A retrospective study conducted at the Instituto Nacional de Investigação em Saúde, Angola, found MDR TB in 38 (50%) of 76 patients with previously untreated TB who attended private and public health services in Luanda (*8*). Discrepancies with previous data might be caused by the different settings (rural vs. urban), study designs (prospective vs. retrospective), population (previously infected included or not), and diagnostic methods (GeneXpert vs. BACTEC [BD, https://www.bd.com]).

Because we collected data from a single center, our results should not be generalized as prevalence in Luanda or all of Angola. Despite that limitation, our finding of higher prevalence (8%) of RR/MDR TB in new TB cases compared with the estimates from WHO, together with the existence of pre-XDR TB cases, calls for a nationwide prevalence survey to strengthen epidemiologic monitoring of TB drug resistance in Angola.
